# A Mini Review on Microcystins and Bacterial Degradation

**DOI:** 10.3390/toxins12040268

**Published:** 2020-04-21

**Authors:** Isaac Yaw Massey, Fei Yang

**Affiliations:** Department of Occupational and Environmental Health, Xiangya School of Public Health, Central South University, Changsha 410078, China; mriymassey@csu.edu.cn

**Keywords:** microcystins, toxicity and carcinogenicity, bacterial degradation, degrading mechanism

## Abstract

Microcystins (MCs) classified as hepatotoxic and carcinogenic are the most commonly reported cyanobacterial toxins found in the environment. *Microcystis* sp. possessing a series of MC synthesis genes (mcyA-mcyJ) are well documented for their excessive abundance, numerous bloom occurrences and MC producing capacity. About 246 variants of MC which exert severe animal and human health hazards through the inhibition of protein phosphatases (PP1 and PP2A) have been characterized. To minimize and prevent MC health consequences, the World Health Organization proposed 1 µg/L MC guidelines for safe drinking water quality. Further the utilization of bacteria that represent a promising biological treatment approach to degrade and remove MC from water bodies without harming the environment has gained global attention. Thus the present review described toxic effects and bacterial degradation of MCs.

## 1. Introduction

Cyanobacteria are organisms that inhabit surface and bottom water. These organisms can accumulate to form blooms and scums which are mostly found on the water surface. WHO [1] reported that blooms of toxic cyanobacteria are gradually increasing worldwide in both frequency and severity. While cyanobacterial blooms occur naturally in water bodies, the combination of environmental factors such as nutrients (nitrogen and phosphorus), weather conditions, carbon dioxide, water body, salinity, sunlight, pH, heavy metals, brief periods of drought and heavy rain may trigger for the proliferation of the blooms [2,3,4,5,6]. Cyanobacterial blooms have become a serious global environmental problem in both developing and developed countries due to the unpleasant odor, taste and cyanobacterial toxins produced. The presence of these toxins may reduce water quality, accumulate and magnify in food chains, and bring about significant negative effects on human health and animals [7,8,9,10]. Of the numerous cyanobacterial toxins discovered, microcystins (MCs) are classified as hepatotoxic and potentially carcinogenic [11], most often present in water [12] and extensively studied in terms of degradation and removal [13,14,15,16,17]. Thus this review focuses on toxic effects and bacterial degradation of MCs.

## 2. Microcystins

### 2.1. Microcystins Synthesis

Microcystins are cyclic heptapeptide hepatotoxins primarily found in marine and freshwaters worldwide [8,9,18,19]. Metcalf et al. [20] also indicated that MCs can be produced in desert environments. More than 30 cyanobacterial species are capable to produce MCs [21]. Interestingly much attention has been given to *Microcystis* sp. owing to its extreme abundance, frequent bloom occurrences and ability to generate MCs [18,22,23,24,25]. *Microcystis* sp. possess a series of MC synthesis genes (mcyA-mcyJ), where mcyA, mcyB and mcyC genes are usually used in detecting toxigenic cyanobacteria [18,26,27]. Thus the ability for cyanobacteria to produce MC is determined by the mcy cluster. During the growth, MCs are retained in cyanobacterial cells and are found to be released during senescence and breakdown processes [28,29,30].

### 2.2. Chemical Properties

**Figure 1:**
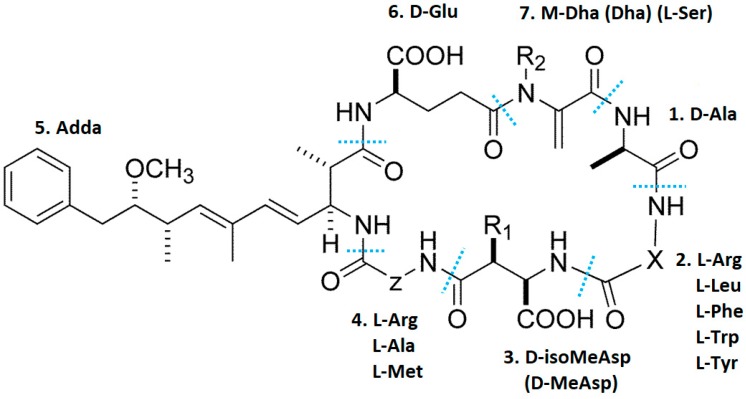
General structure of hepatotoxic cyclic peptides, microcystins. 1–7 signify seven amino acid residues. X and Z in positions two and four are highly variable L-amino acids (reproduced from [35], 2019, MDPI).

Approximately 246 variants of MC have been characterized, which exhibit different degrees of toxicity [31]. Of the numerous MC variants characterized, MC-LR, MC-RR and MC-YR are the most frequently found in the environment, very toxic and extensively studied. MC-LF and MC-LW have also been shown with quite high concentrations [14,18,32,33,34]. In general MCs share a common cyclic structure cyclo-(-D-Ala-L-X-D-MeAsp-L-Z-Adda-D-Glu-Mdha) (Figure 1) which makes them capable to resist physical and chemical factors. X and Z represent highly variable amino acids, D-MeAsp represents D-erythro-b-methylaspartic acid, Adda represents (2S, 3S, 8S, 9S) 3- amino-9 methoxy-2,6,8-trimethyl-10-phenyldeca-4, 6-dienoic acid and Mdha represents N-methyldehydroalanine. The unique structure Adda is crucial for biological activity of MC molecules [8]. Further the variants of MC mainly differ in X and Z amino acids, and methylation or demethylation on MeAsp and Mdha. For instance MC-LR is known to contain Leucine (L) and Arginine (R) amino acids, MC-RR consists of two ‘R’ amino acids and MC-YR contains Tyrosine (Y) and ‘R’ amino acids [8].

The cyclic structure of MCs is noted to be responsible for their stability in temperature and pH. MCs are capable to survive at extreme temperatures greater than 300 °C, low temperatures without sunlight and dryness, and extreme high and low pH [36,37,38]. MCs are documented to have a size of approximately 3 nm in diameter and molecular weight ranging between 800 and 1100 Daltons, which primarily depends on the amino acid composition mainly at variable positions and modifications on the incorporated amino acids [39]. Although MCs are known mainly to be polar molecules, some variants contain hydrophobic amino acid residues in the highly variable parts of the molecules. For instance, MC-LF and MC-LW, the more hydrophobic phenylalanine (F) and tryptophan (W), respectively, have replaced ‘R’ in MC-LR. The hydrophilic functional groups of MC-LR include carboxyl groups on glutamic and methylaspartic acid and the amino group on ‘R’, while the Adda residue is hydrophobic [40]. In addition the net charge of MC-LR was noted to be negative (−1) at most pH values (3 < pH < 12), as the net result of the dissociation of two carboxyl groups and single positive charge of the amino group [41].

### 2.3. Toxicity and Carcinogenicity

Microcystins have shown humans and animals toxicity [9,21,42,43] with lethal dose LD_50_ by the intraperitoneal route ranging from 50 (MC-LR) to 600 (MC-RR) µg/kg and oral LD_50_ of 5000 µg/kg as indicated in mice [8]. The primary mechanism of MCs toxicity is the inhibition of protein phosphatases (PP1) and protein phosphatases (PP2A). This may lead to hyperphosphorylation of key cellular proteins, hepatic hemorrhage, necrosis, inflammation, apoptosis, cytoskeletal and DNA destruction [44,45]. MCs may also induce oxidative stress to trigger cellular apoptosis, destabilize cytoskeleton, enhance cancer cell invasion and damage DNA [46,47].

Microcystins are able to get into the mammalian body through consumption of contaminated water, food and algal dietary supplements, body contact, hemodialysis and in a lesser extent inhalation [9]. Exposure to MCs may show liver toxicity which is the main target organ. Mice exposed to MC-LR had noteworthy rise in clinical chemistry parameters alanine aminotransferase, aspartate aminotransferase, alkaline phosphatase, total bilirubin, and cholesterol, with significant increase in females compared to males. In addition toxic manifestations near the central veins as well as mid lobular areas were observed [48]. At low MC-LR concentration via waterborne exposure, the fish *Geophagus brasiliensis* exhibited alteration on the liver antioxidant system and histopathologies such as dilation of sinusoids and vacuolization of hepatocytes were evident [49]. Report also indicated that over 80% of the small population of fishers who lived for many years on Meiliang Bay of Lake Taihu had at least one abnormal serum marker and the serum biochemical indices of liver function including aspartate aminotransferase (AST)/alanine aminotransferase (ALT), triglyceride (TG), globulin (GLB) and lactate dehydrogenase (LDH) revealed liver damage and lipid metabolism dysfunction due to the close positive association with MC contamination [50]. Interestingly the long-term exposure of MC-LR on mice liver also showed hepatic steatosis with molecular alterations in circadian rhythm regulation, lipid metabolic processes, and the cell cycle pathway. Further, at or above the no-observed adverse effect level (NOAEL), MC-LR exposure worsened the pathological phenotype towards nonalcoholic steatohepatitis disease (NASH) or fibrosis [50]. It is worth knowing that other organs including respiratory, renal, cardiovascular, intestinal, central nervous, and reproductive system may also be affected due to the organic anion transporting polypeptides (OATPs) which actively transport MCs into cells [35,51]. The chronic low-dose of MC-LR exposure resulted to alveolar collapse, lung cell apoptosis and breach of cell junction integrity. ATII cells were also capable to uptake MC-LR and induced apoptosis and disrupted cell junction integrity [52]. Chronic oral administration of MC-LR also resulted in mitochondrial DNA (mtDNA) neuron damage and histopathological abnormalities as well as mtDNA damage were evident in the hippocampus and cerebral cortex with distinct effects on these two brain regions [53]. Further exposure to MC-LR damaged the microstructure of the jejunum and expression levels of inflammation-related factors interleukin (IL)-1β, interleukin (IL)-8, tumor necrosis factor alpha (TNF-α), as well as transforming growth factor-β1 (TGF-β1), and interleukin (IL)-10 were altered at different MC-LR concentrations [42]. A slight change in serum creatinine (SCr) levels, clear decrease in blood urea nitrogen (BUN) levels, enlarged renal corpuscles and widened of kidney tubules, lymphocyte infiltration in the interstitial tissue, as well as renal pelvis of mice kidney were found after chronic oral MC-LR exposure in mice [54]. In addition, entry of MC-LR in male *Macrobrachium rosenbergii* testis down-regulated hemolymph testosterone (T) levels, damaged testicular germ cells, mitochondria as well as cell junctions, inhibited testicular development and significantly induced the expression of gonadal development related genes in the testis and eyestalk [55]. In zebra fish larvae, MC-LR was noted to cause angiodysplasia, destroy vascular structures and decrease lumen size which triggered a decline of the blood flow area in the blood vessels and brain hemorrhage. Further in the human umbilical vein endothelial cells (HUVECs), varying MC-LR concentrations promoted apoptosis and activated caspase 3/9, increased the level of mitochondrial reactive oxygen species (ROS) and reduced mitochondrial membrane potential. MC-LR also fostered the expression of p53 and inhibited the expression of PCNA [56]. The evidence thus signifies toxic potential of low-dose or chronic exposure to MCs can cause severe chronic injuries or significantly threaten the various organs of mammals and pose potential carcinogenic effects. Therefore people with long-term exposure to MCs may be at a higher risk of developing various diseases including nonalcoholic fatty liver disease (NAFLD), NASH, bronchial tubes, gastrointestinal disorders, inflammatory intestinal disease, Alzheimer disease, acute kidney failure, hypertrophic cardiomyopathy and death.

A number of human and animal fatalities and severe poisonings have been attributed to MC-containing *Microcystis* blooms [9,21,57,58]. MCs were noted to affect the growth and physiological functions of aquatic and terrestrial animals, livestock, pets and wildlife due to their bioaccumulation of these toxins. Moreover MCs were transferred along the food chain and intoxicated other organisms [57]. In Egypt, MCs (free and bound forms) monitored in tilapia fish from three tropical fishponds containing high concentrations of MCs were observed in the tilapia fish intestines, livers and edible tissues, and it was estimated to impose significant negative health consequences on human and other organisms when consumed [59]. A similar investigation was carried out by Greer et al. [60] from aquaculture farms in Southeast Asia and reported the presence of high MCs concentration in the liver and muscle tissue of tilapia fish, which represented a health risk when consumed. Bearing this in mind, further monitoring of MCs, aquaculture farming, fish and beyond is vital to ensure safe water and food for mammalian consumption. In addition, no catching of fish from fishponds during cyanobacterial blooms active safety policies and guidelines to safeguard human health implementations should be ensured and abide. One of the most severe cases of human poisoning occurred in Caruaru, Brazil, in February 1996 when a bloom of *Microcystis* in a drinking water reservoir contaminated the water supply of a hemodialysis center with MC-LR resulting to 131 patient casualties [58].

It is worth noting that the presence of MCs mainly resulting from irrigation water may also induce plant and crop inhibition of these toxins to reduce yield, poison food and pose high ecological risk. MCs accumulation in roots was found to inhibit growth and further decreased photosynthetic rate and chlorophyll content in rice after irrigation with water contaminated with MCs [61]. The presence of MC-LR and dmMC-LR was noted in fruits of pepper *Capsicum annuum* and was found to affect antioxidant systems in the fruits after irrigation with contaminated MC water [62]. Further lettuce plants irrigated with MCs contaminated water from the seed and cotyledons stage exhibited higher photosynthetic capacity, chlorophylls as well as leaf nitrogen content, and significant MCs accumulation was observed in various lettuce tissues, constituting a serious public health risk when utilized [63]. Cucumber plants irrigated with MCs extraction contaminated water also inhibited the growth of cucumber at different growth stages (seedling stage > early flowering stage > fruiting stage), and further affected yield and fruit quality. Interestingly contents of vitamin C, soluble sugar and organic acid in fruits of cucumber at seedling stage were declined [64]. It is of interest that irrigation waters containing MCs (MC-LR, MC-RR, and MC-YR) collected from southern China regions were the major source of MCs accumulation in soils and vegetables, and majority of the vegetables exhibited moderate or high human health risk through diet [65]. The findings imply irrigation with MCs contaminated water is capable of threatening plant growth and human health. Based upon this knowledge, it is essential for human to exercise care when ingesting fruits as part of their diet and also strengthen agricultural irrigation management system through monitoring and controlling of contaminated water irrigation with MCs to avoid harmful accumulation of these toxins, destruction to plant growth and potential high ecological and human health hazards. Due to MC toxic consequences, the International Agency for Research on Cancer (IARC) has categorized this toxin as a possible carcinogen [11]. To lessen and prevent MC health hazards, the World Health Organization (WHO) also suggested a provisional 1 µg/L MC guidelines for drinking water quality [66] and maximum 20,000 cyanobacterial cells mL^−1^ or 10 µg·L^−1^ of chlorophyll-a (where about 2–4 µg·L^−1^ of MCs is expected) guidelines for safe recreational water environment [67].

## 3. Treatment Approach

Microcystins are considered as one of the biggest water pollution problems for global public health due to their ability to induce a range of water safety issues and undesirable health manifestations on animals and humans [8,9,57,66]. Drinking water should be safe enough to be utilized with low risk of immediate or long-term harm. In view of this, it is necessary to monitor the utilization of water for toxic cyanobacteria as well as MCs, and treat them for safety domestic, agricultural and recreational purposes. To treat water contaminated with toxin, the toxin’s physical and chemical properties, nature as well as cyanobacterial growth and blooms patterns should be considered [8]. A large number of drinking water treatment plants utilize conventional and advanced oxidation water treatment processes to treat water contaminated with MCs. Interestingly, these approaches are sometimes considered too expensive to entirely remove a contaminant that occasionally occurs [68]. Ozonation, chlorination, and chloramination treatment approaches which were unable to thoroughly eliminate MCs in reagent-grade water, Colorado river water and California State Project water further generated by products [69]. The solar/chlorine process, chlorination and solar irradiation though were noted to decrease concentration and hepatotoxicity of MC-LR, complete degradation was not obtained [15]. The ozonation of six MCs also realized a decline in the toxicities and concentrations of MC-LR, MC-RR, MC-LA and MC-LF. Nevertheless, MC-YR and MC-LW revealed a gap between the concentration and toxicity due to partial eradication of Adda [13]. Further conventional coagulation and filtration had limited efficiency in eliminating MCs, as demonstrated in treatment plants located in Czech Republic and in some US states (i.e., California, Texas, Oklahoma, Florida, and Vermont) [70,71]. The findings indicates that the conventional and advanced oxidation water treatment processes are generally expensive to use, ineffective at removing and/or destroying MCs and are capable to generate harmful metabolites. Consequently, it is essential to seek for an efficient and a cost-effective treatment approach that will not invoke any potential harmful metabolites after treatment. The use of bacteria represents a recent investigated and promising biological treatment approach for degrading and removing MCs from water bodies without harming the environment.

### 3.1. Biological Degradation by Bacteria

The biological treatment approach that generally requires little or no maintenance has proven to be environmentally-friendly, effective and can be depended upon to eliminate MCs in water sources compared to the conventional treatment approaches. At present, the biological treatment is becoming more useful as MCs can also be removed without the addition of chemicals capable of producing undesirable metabolites [72,73,74,75,76]. Since the first isolation of bacteria strain capable of degrading MC (from the Murrumbidgee river, Australia [77]), various other bacteria strains have successfully been isolated (Table 1). These bacteria mainly belong to Proteobacteria (α, β and γ), Actinobacteria and Bacilli.

In practice majority of the MC-degrading bacteria are limited to the genus *Sphingomonas* [76,104,105]. Other species among the genera *Acinetobacter*, *Arthrobacter*, *Bacillus*, *Novosphingobium*, *Paucibacter*, *Pseudomonas*, *Sphingopyxis* and *Stenotrophomonas* have also been reported for MCs degradation (Table 1). *Bacillus* sp. AMRI-03 completely degraded MC-RR within five days after a lag period of two days [83]. *Arthrobacter* sp. C6, F7, F10, R1, R4, R6 and R9 demonstrated MC-LR removal within 72 hr [81,82]. Also within 24 days, *Pseudomonas aeruginosa* DMXS eliminated [D-Leu^1^] MC-LR [75]. *Novosphingobium* sp. KKU-25s also decomposed [Dha(7)]MC-LR within 24 h [96]. Further *Stenotrophomonas maltophilia* 4B4 showed total MC-LR removed within 10 days while MC-RR and MC-LF elimination occurred within 12 and 14 days respectively [16]. In a recent publication, a novel bacteria strain YF1 of the genus *Sphingopyxis* indicated thorough MC-LR degradation within 120 min [123]. The evidence thus indicates that bacteria strains isolated from many different environmental habitats around the world have strong MC-degrading ability, and may play a significant role in the natural degradation and removal of MCs.

The combination of two or more bacteria strains (bacterial community) have also proven to be capable of degrading MC-LR (Table 1). The seven isolates *Acinetobacter* sp., *Hyphomicrobium aestuarii*, *Pseudoxanthomonas* sp., *Rhizobium* sp., *Sphingobium* sp., *Sphingomonas* sp. and *Steroidobacter* sp. from Taiwan reservoir, China [130] and 10 isolates *Acinetobacter* sp., *Aeromonas* sp., *Novosphingobium* sp., *Ochrobactrum* sp., *Pseudomonas* sp., *Rhodococcus* sp., *Sphingomonas* sp., *Sphingopyxis* sp., *Stenotrophomonas* sp. and *Steroidobacter* sp. from drinking water reservoir in Southern California [126] successfully indicated complete MC-LR degradation. Moreover bacterial community isolated from natural waters with previous cyanobacterial contamination was noted to entirely removal MC-LR without lag phase, however, the composition of the bacterial community was not analyzed [134]. In a recent study, acclimatized-TSFU bacterial community comprising of novel strains *Chryseobacterium* sp. and *Pseudomonas fragi* was also found to completely degrade MC-LR [127]. The natural bacterial community mainly consisting of *Agrobacterium* sp., *Bosea* sp., *Brevundimonas* sp., *Hyphomicrobium* sp., *Rasbo* sp., *Rhizobium* sp., *Rhodococcus* sp., *Roseomonas* sp., *Mesorhizobium* sp., *Nitrosococcus* sp., *Sandaracinobacter* sp. and *Sphingomonas* sp. isolated from the mucilage of *M. aeruginosa* colonies during a bloom in a French pond completely degraded MC-LR and Des-MCLR. It is of interest that the bacterial community also degraded cyanobacterial secondary metabolites such as cyanopeptolins and aerucyclamides [131]. The findings of this study suggest that, bacterial community may possess the ability not only to degrade MC but also other cyanobacterial secondary metabolites. At present only a few MC-degrading bacterial communities have been obtained for MC-LR degradation. Further studies are required to investigate the degrading ability of bacterial community on other MC variants.

### 3.2. Enzymatic Mechanisms of Microcystins Biodegradation

A growing number of novel bacteria strains with the ability to degrade MCs are being revealed. However, only one metabolic pathway liable for degrading these toxins and is encoded by the *mlr* gene cluster is fully described. A novel pathway involving four genes (*mlrABCD*), three intracellular hydrolytic enzymes (MlrABC) and two intermediate products; linearized MC-LR (Adda-Glu-Mdha-Ala-Leu-Masp-Arg-OH) and tetrapeptide (Adda-Glu-Mdha-Ala-OH) for MC-LR degradation using *Sphingomonas* sp. ACM- 3962 was demonstrated [105]. The MlrA enzyme encoded by *mlrA* gene cleaved the Adda-Arg peptide bond of MC-LR, which converted MC-LR to linearized MC-LR. The MlrB enzyme encoded by *mlrB* gene hydrolyzed the Ala–Leu bond, converting the linearized MC-LR to a tetrapeptide. The MlrC enzyme encoded by *mlrC* gene broke the tetrapeptide into smaller peptides and amino acids. Transport of MC and its degradation products were assumed to be facilitated via *mlrD* gene [135]. Nevertheless, these smaller peptides and amino acids were not well described. In subsequent investigations, Imanishi et al. [106] and Harada et al. [107] showed products of MC-LR degradation which were consistent with the previous report [105,135] and further isolated Adda using *Sphingomonas* sp. B-9. Successive studies have also confirmed the existence of *mlr* cluster components in other MC-degrading bacteria species and strains (as depicted in Table 1).

**Figure 2:**
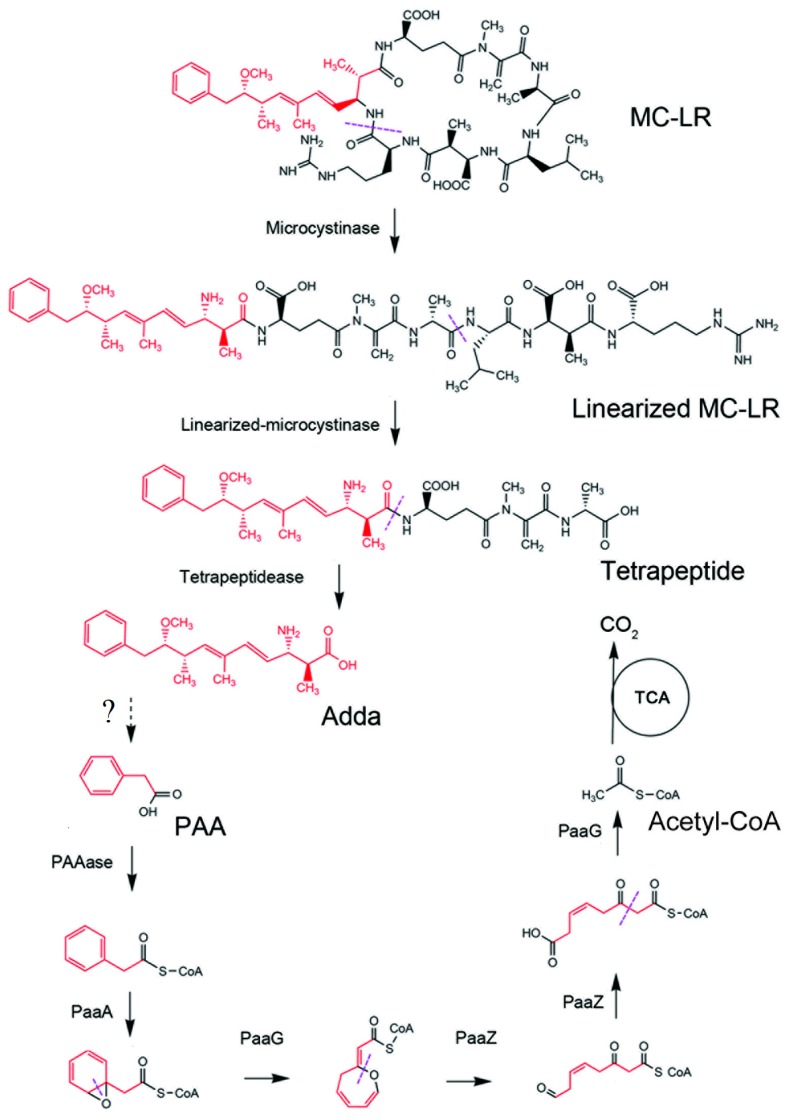
Biodegradation pathway of MC-LR by *Sphingopyxis* sp. YF1 (reproduced from [123], 2020, Elsevier Ltd.).

Regarding the sequential enzymatic hydrolyses of peptide bonds, *mlrC* gene was found not only to act on the tetrapeptide but is also capable to hydrolyze linearized MC-LR without earlier processing by *mlrB* gene [114,136]. *Sphingopxyis* sp. USTB-05 showed complete MCs (MC-LR, MC-RR and MC-YR) degradation with different intermediate products [30,118,119,120,121]. These findings suggest that the enzymatic processes and degradation pathways may vary between MC variants and bacteria strains. Further aside the known MC-LR degradation products (linearized MC-LR, tetrapeptide and Adda), Ding et al. [116] identified eight new different intermediate degradation products namely, three tripeptides (Adda-Glu-Mdha, Glu-Mdha-Ala, and Leu-MeAsp-Arg), three dipeptides (Glu-Mdha, Mdha-Ala, and MeAsp-Arg) and two amino acids (Leu, and Arg) using *Sphingopxyis* sp. m6. These findings also suggest that more than three intermediate products are capable to occur during MC degradation. In a recent study Yang et al. [123] found a potential link between *paa* gene clusters for phenylacetic acid (PAA) degradation in the neighborhoods of *mlrABCD* gene cluster. Interestingly complete MC-LR degradation by *Sphingopyxis* sp. YF1 without Adda as the final degradation product (as illustrated in Figure 2.) was noted. MC-LR was sequentially degraded into linearized MC-LR, tetrapeptide and Adda. The Adda was further degraded into PAA, and was converted into PAA-CoA by PAAase. Subsequently PAA-CoA was degraded to acetyl coenzyme A (acetyl-CoA) by PaaA, PaaG and PaaZ homologues. Finally, acetyl-CoA was completely converted to CO_2_ through tricarboxylic acid (TCA) cycle [123]. The evidence suggests that PAA may be the downstream metabolism path of MC degradation. However, further study needs to determine the kind of enzyme that degraded Adda into PAA.

It is well established that the degradation pathways and enzymatic processes in α, β and γ Proteobacteria strains among the genera *Bordetella*, *Sphingomonas*, *Sphingopxyis Stentophomonas* and *Rhizobium* [17,73,89,104,106,111,113,115,117,124] as well as bacterial communities including *Acinetobacter* sp., *Hyphomicrobium aestuarii*, *Pseudoxanthomonas* sp., *Rhizobium* sp., *Sphingobium* sp., *Sphingomonas* sp. and *Steroidobacter* sp. (Indigenous bacterial mixed culture) and *Chryseobacterium* sp. and *Pseudomonas fragi* (Acclimatized-TSFU) [127,130] are well described. These bacteria strains and bacterial communities found to degrade different variants of MC contained the *mlr* homologues genes, and linearized MC, tetrapeptide and/or Adda were evident as intermediate products. At present only the *mlrA* gene has been observed in *Bacillus* sp. [83,84,86]. Although the *Bacillus* sp., *Paucibacter* sp., *Pseudomonas* sp. and *Ralstonia solanacearum* as well as bacterial communities including *Klebsiella* sp. and *Stenotrophomonas* sp. (YFMCD1), *Alcaligenes faecalis* and *Stenotrophomonas acidaminiohila* (YFMCD4) and *Bordetella* sp., *Burkholderia* sp., *Cupriavidus* sp., *Methylotenera* sp., *Polaromonas* sp., *Polynucleobacter* sp., *Ralstonia* sp. and *Variovorax* sp. (Bacterial community) (as depicted in Table 1) also degraded different variants of MC, the degradation pathways and/or enzymatic processes were inadequately described. Clearly, further investigation needs to elucidate the MC-degrading metabolism of these strains and communities. It is of interest that the MC degradation mechanism for *Acinetobacter* sp., *Aeromonas* sp., *Arthrobacter* sp., *Bifidobacterium longum*, *Brevibacterium* sp., *Burkholderia* sp., *Lactobacillus rhamnosus*, *Methylobacillus* sp., *Morganella morganii*, *Ochrobactrum* sp. and *Rhodococous* sp. as well as bacterial communities including *Acinetobacter* sp., *Aeromonas* sp., *Novosphingobium* sp., *Ochrobactrum* sp., *Pseudomonas* sp., *Rhodococcus* sp., *Sphingomonas* sp., *Sphingopyxis* sp., *Stenotrophomonas* sp. and *Steroidobacter* (Bacterial consortia) and *Microbacterium* sp. and *Rhizobium* sp. (Bacterial consortium) (as shown in Table 1) also need to be identified and clarified. It is worth noting that the *Enterobacter* sp. YF3, *Acidovorax facilis* Lew-2, *Bacillus thuringiensis* LEw-2010, *Brevibacillus brevis* LEw-1238, *Pseudomonas putida* LEw-1033 and LEw-2166, *Stenotrophomonas maltophila* LEw-1278 and *Bacillus* sp. [72,74,87] demonstrated MC-LR degradation independent of the MC-degrading *mlrABCD* genes. These findings indicate that MC degradation may not necessarily follow the pathways of the *mlr* degrading mechanism.

## 4. Conclusions

In this current paper, toxic effects and bacterial degradation of MCs were reviewed. Studies indicate that the cyclic heptapeptide hepatotoxins MCs are primarily produced by species of *Microcystis* containing a series of MC synthesis genes. MCs inhibit protein phosphatases and constitute a natural health hazards in the environment. MCs are chemically stable in water and conventional water treatment approaches have failed to completely remove them. MC toxic effect led to the establishment of WHO’s 1 µg/L MC guidelines for safe drinking water quality. To provide safe drinking water, biological degradation is considered the most successful solution for MCs removal in the natural environment. Bacteria identification for different variants of MC degradation has been reported from various environmental habitats worldwide. The metabolism of these bacteria showed four genes (*mlrABCD*), three intracellular hydrolytic enzymes (MlrABC) and three degrading products (linearized MC-LR, tetrapeptide and Adda). However studies about *Sphingopxyis* that resulted to a hydrolytic pathway via the discovery of eight new intermediate products, identification of different intermediate products from varying MC variants and the downstream MC degradation route by PAA metabolism which identified CO_2_ as the final degrading product have paved way for additional investigations on the biodegradation mechanism of MCs. Further studies need to explore the PAA metabolism on other MC degrading bacteria strains. Moreover, whether any degradation products exist between Adda and PAA, how the degrading product can be converted into PAA, and the functional genes as well as proteins involved in the metabolism process, need to be determined.

## Figures and Tables

**Table 1:** Microcystin degrading bacteria.

Species	Strain	*mlr* Gene	Degradable MC Variant	Class Affiliated	Reference
*Acidovorax facilis*	LEw-2		MC-LR	β-proteobacteria	[74]
*Acinetobacter* sp.	CMDB-2		MC-LR	γ-proteobacteria	[78]
	WC-5		MC-LR, MC-RR	γ-proteobacteria	[79]
*Aeromonas* sp.			MC-LR	γ-proteobacteria	[80]
*Arthrobacter* sp.	C6		MC-LR, MC-LW, MC-RR, MC-LF, MC-LY	Actinobacteria	[81,82]
	F7		MC-LR, MC-LW, MC-RR, MC-LF, MC-LY	Actinobacteria	[81,82]
	F10		MC-LR	Actinobacteria	[82]
	R1		MC-LR	Actinobacteria	[82]
	R4		MC-LR, MC-LW, MC-RR, MC-LF, MC-LY	Actinobacteria	[81,82]
	R6		MC-LR	Actinobacteria	[82]
	R9		MC-LR	Actinobacteria	[82]
*Bacillus* sp.	AMRI-03	*mlrA*	MC-RR	Bacilli	[83]
	EMB	*mlrA*	MC-LR, MC-RR	Bacilli	[84]
	JZ-2013		MC-LR	Bacilli	[85]
	LEw-2010		MC-LR	Bacilli	[74]
	SSZ01	*mlrA*	MC-RR	Bacilli	[86]
			MC-LR	Bacilli	[87]
*Bifidobacterium longum*	Bb12		MC-LR	Actinobacteria	[88]
	46		MC-LR	Actinobacteria	[88]
	420		MC-LR	Actinobacteria	[88]
*Bordetella* sp.	MC-LTH1	*mlrA*	MC-LR, MC-RR	β-proteobacteria	[89]
*Brevibacillus brevis*	LEw-1238		MC-LR	Bacilli	[74]
*Brevibacterium* sp.	F3		MC-LR, MC-LW, MC-RR, MC-LF, MC-LY	Actinobacteria	[81]
*Burkholderia* sp.			MC-LR, [D-Leu^1^]MC–LR	β-proteobacteria	[90]
*Enterobacter* sp.	YF3		MC-LR	γ-proteobacteria	[72]
*Lactobacillus rhamnosus*	GG		MC-LR	Bacilli	[88]
	LC-705		MC-LR	Bacilli	[88]
*Lysinibacillus boronitolerans*	CQ5		MC-LR	Bacilli	[91]
*Methylobacillus* sp.	J10		MC-LR, MC-RR	β-proteobacteria	[92]
*Morganella morganii*	C25216		MC-LR	Actinobacteria	[93]
	C25217		MC-LR	Actinobacteria	[93]
	C25220		MC-LR	Actinobacteria	[93]
*Novosphingobium* sp.	KKU-12		[Dha^7^] MC-LR	α-proteobacteria	[94]
	KKU15		[Dha^7^] MC-LR	α-proteobacteria	[95]
	KKU-25s	*mlrABCD*	[Dha^7^] MC-LR	α-proteobacteria	[96]
	THN-1	*mlrABCD*	MC-LR	α-proteobacteria	[97]
*Ochrobactrum* sp.	FDT5		MC-LR	α-proteobacteria	[98]
*Paucibacter* sp.	CH		MC-LR	β-proteobacteria	[99]
	IM-4		MC-LR, MC-RR, MC-YR	β-proteobacteria	[100]
	2C20^T^		MC-LR, MC-RR, MC-YR	β-proteobacteria	[101]
*Pseudomonas* sp.	DMXS		[D-Leu^1^] MC-LR	γ-proteobacteria	[75]
	LEw-1033		MC-LR	γ-proteobacteria	[74]
	LEw-2166		MC-LR	γ-proteobacteria	[74]
	Pseudomonas aeruginosa		MC-LR	γ-proteobacteria	[102]
	WC-4		MC-LR, MC-RR	γ-proteobacteria	[79]
*Ralstonia solanacearum*			MC-LR, MC-RR	β-proteobacteria	[103]
*Rhizobium* sp.	TH	*mlrABCD*	MC-LR	α-proteobacteria	[73]
*Rhodococcus* sp.	C1		MC-LR, MC-LW, MC-RR, MC-LF, MC-LY	Actinobacteria	[81]
	C3		MC-LR	Actinobacteria	[81,82]
*Sphingomonas* sp.	ACM-3962/MJ-PV	*mlrABCD*	MC-LR, MC-RR	α-proteobacteria	[77,104,105]
	B9	*mlrA*	MC-LR, MC-RR, 3-DMMCLR, DHMCLR, MCLR-Cys	α-proteobacteria	[106,107,108]
	CBA4		MC-RR	α-proteobacteria	[109]
	MD-1	*mlrABCD*	MC-LR, MC-RR, MC-YR	α-proteobacteria	[104]
	MDB2			α-proteobacteria	[110]
	MDB3			α-proteobacteria	[110]
	Y2	*mlrA*	MC-LR, MC-RR, MC-YR	α-proteobacteria	[111]
	7CY		MC-LR, MC-LW, MC-RR, MC-LF, MC-YR, MC-LY	α-proteobacteria	[112]
*Sphingopyxis* sp.	a7	*mlrACD*	MC-LR	α-proteobacteria	[76]
	C1	*mlrABC*	MC-LR	α-proteobacteria	[113,114]
	IM-1	*mlrABCD*	MC-LR, MC-RR, MC-YR	α-proteobacteria	[100]
	IM-2	*mlrABCD*	MC-LR, MC-RR, MC-YR	α-proteobacteria	[100]
	IM-3	*mlrABCD*	MC-LR, MC-RR, MC-YR	α-proteobacteria	[100]
	LH21	*mlrABCD*	MC-LR, MC-LA	α-proteobacteria	[115]
	m6	*mlrABCD*	MC-LR	α-proteobacteria	[116]
	MB-E	*mlrABCD*	MC-LR, MC-LW, MC-YR, MC-LY, MC-LF	α-proteobacteria	[17]
	TT25	*mlrA*	MC-LR, MC-RR, MC-YR	α-proteobacteria	[117]
	USTB05	*mlrABCD*	MC-LR, MC-RR, MC-YR	α-proteobacteria	[30,118,119,120,121]
	X20	*mlrABCD*	MC-LR	α-proteobacteria	[122]
	YF1	*mlrABCD*	MC-LR	α-proteobacteria	[123]
*Stenotrophomonas* sp.	EMS	*mlrA*	MC-LR, MC-RR	γ-proteobacteria	[124]
	LEw-1278		MC-LR	γ-proteobacteria	[74]
	MC-LTH2		MC-LR, MC-RR	γ-proteobacteria	[125]
	4B4	*mlrABCD*	MC-LR, MC-RR, MC-LW, MC-LF	γ-proteobacteria	[16]
*Acinetobacter* sp.	Bacterial		MC-LR	γ-proteobacteria	[126]
*Aeromonas* sp.	consortia			γ-proteobacteria	
*Novosphingobium* sp.				α-proteobacteria	
*Ochrobactrum* sp.				α-proteobacteria	
*Pseudomonas* sp.				γ-proteobacteria	
*Rhodococcus* sp.				Actinobacteria	
*Sphingomonas* sp.				α-proteobacteria	
*Sphingopyxis* sp.				α-proteobacteria	
*Stenotrophomonas* sp.				γ-proteobacteria	
*Steroidobacter* sp.				γ-proteobacteria	
*Chryseobacterium* sp.	TSFU	*mlrABC*	MC-LR	Flavobacteriia	[127]
*Pseudomonas fragi*				γ-proteobacteria	
*Alcaligenes faecalis*	YFMCD4		MC-LR	β-proteobacteria	[128]
*Stenotrophomonas acidaminiohila*				γ-proteobacteria	
*Klebsiella* sp.	YFMCD1		MC-LR	γ-proteobacteria	[129]
*Stenotrophomonas* sp.				γ-proteobacteria	
*Acinetobacter* sp.	Indigenous	*mlrAD*	MC-LR	γ-proteobacteria	[130]
*Hyphomicrobium aestuarii*	bacterial			α-proteobacteria	
*Pseudoxanthomonas* sp.	mixed			γ-proteobacteria	
*Rhizobium* sp.	culture			α-proteobacteria	
*Sphingobium* sp.				α-proteobacteria	
*Sphingomonas* sp.				α-proteobacteria	
*Steroidobacter* sp.				γ-proteobacteria	
*Agrobacterium* sp.	Natural		MC-LR, Des-MCLR	α-proteobacteria	[131]
*Bosea* sp.	bacterial			α-proteobacteria	
*Brevundimonas* sp.	community			α-proteobacteria	
*Hyphomicrobium* sp.				α-proteobacteria	
*Rasbo* sp.				α-proteobacteria	
*Rhizobium* sp.				α-proteobacteria	
*Rhodococcus* sp.				Actinobacteria	
*Roseomonas* sp.				α-proteobacteria	
*Mesorhizobium* sp.				α-proteobacteria	
*Nitrosococcus* sp.				γ-proteobacteria	
*Sandaracinobacter* sp.				α-proteobacteria	
*Sphingomonas* sp.				α-proteobacteria	
*Bordetella* sp.	Bacterial	*mlrABCD*	MC-LR	β-proteobacteria	[132]
*Burkholderia* sp.	community			β-proteobacteria	
*Cupriavidus* sp.				β-proteobacteria	
*Methylotenera* sp.				β-proteobacteria	
*Polaromonas* sp.				β-proteobacteria	
*Polynucleobacter* sp.				β-proteobacteria	
*Ralstonia* sp.				γ-proteobacteria	
*Variovorax* sp.				β-proteobacteria	
*Microbacterium* sp.	Bacterial		MC-LR	Actinobacteria	[133]
*Rhizobium* sp.	consortium			α-proteobacteria	

MC: Microcystin; Degradable MC variant: MC variant that can be degraded by the corresponding bacteria strain; *mlr* gene: A gene cluster that plays a crucial role in the sequential enzymatic hydrolyses of peptide bonds.
